# Splenic Artery Pseudoaneurysm as a Complication of Chronic Pancreatitis

**DOI:** 10.7759/cureus.75025

**Published:** 2024-12-03

**Authors:** Oscar Antonio Regalado Morales, Luis Alberto Solís García, José Luis Herrera Alanís, Alexis Fernando Chávez Silva, Samuel Iván Espinoza Tristán

**Affiliations:** 1 Radiology, Hospital Regional Instituto de Seguridad y Servicios Sociales de los Trabajadores del Estado (ISSSTE), Monterrey, MEX

**Keywords:** abdominal computed tomography, abdominal radiology, chronic pancreatitis (cp), pseudoaneurysm of splenic artery, vascular lesions

## Abstract

Splenic artery pseudoaneurysms represent one of the most feared vascular complications of chronic pancreatitis. Sectional imaging studies such as computed tomography represent the first-line diagnostic tool for this pathology, being found as an incidental finding in patients with risk factors. We report the case of a splenic artery pseudoaneurysm in a 55-year-old patient diagnosed with chronic pancreatitis.

## Introduction

Pancreatitis is one of the main causes of acute abdomen worldwide. The acute form represents an inflammatory disease secondary to the intraparenchymal activation of pancreatic enzymes, which can be divided into edematous and necrotizing according to the findings in imaging studies [1]. Chronic pancreatitis represents an entity characterized by fibrosis, chronic inflammation, and loss of acinar cells. The main clinical findings are chronic abdominal pain, episodes of acute pancreatitis, and endocrine insufficiency [2].

Both forms of pancreatitis can present complications during the evolution of the clinical picture, which can be systemic and local. Within the latter, vascular complications are the most feared, but fortunately rare, presenting as thrombosis and the formation of pseudoaneurysms of visceral vessels [3].

In this study, we report the case of a patient with a history of chronic pancreatitis who presented a pseudoaneurysm of the splenic artery.

## Case presentation

**Figure 1:**
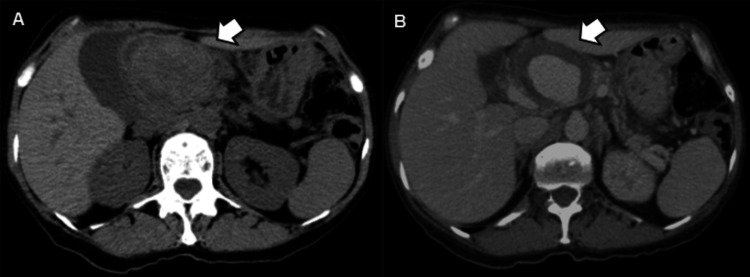
Computerized tomography of the abdomen in soft tissue window in (A) simple and (B) portal venous phase. (A) This image shows a rounded, partially defined, and heterogeneous pre-aortic lesion in the upper abdomen. (B) Following intravenous contrast injection, there is a central and homogeneous enhancement, suggestive of partially thrombosed pseudoaneurysm.

**Figure 2:**
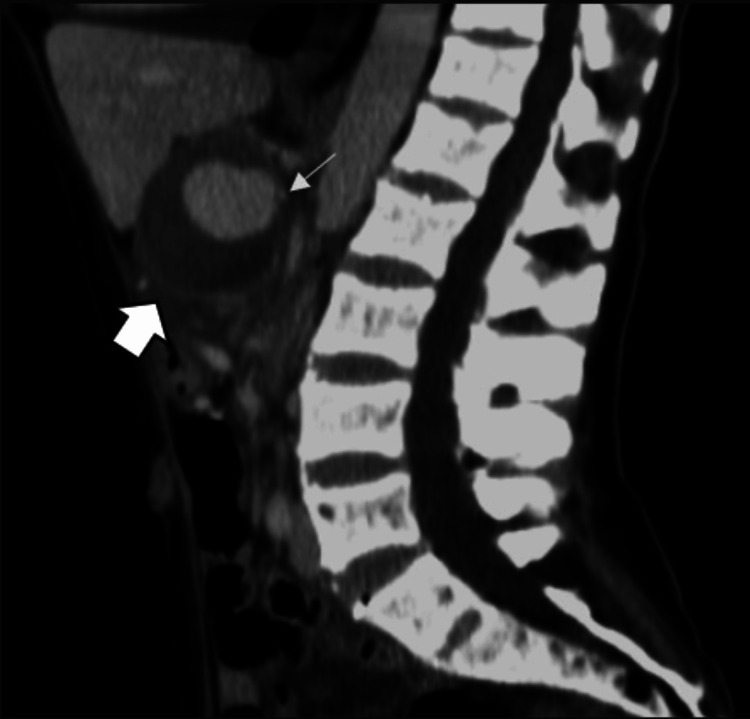
Computerized tomography in sagittal reconstruction during the portal venous phase. This image shows the lesion (thick arrow) adjacent to the hepatic hilum in contact with the spleen artery (thin arrow).

**Figure 3:**
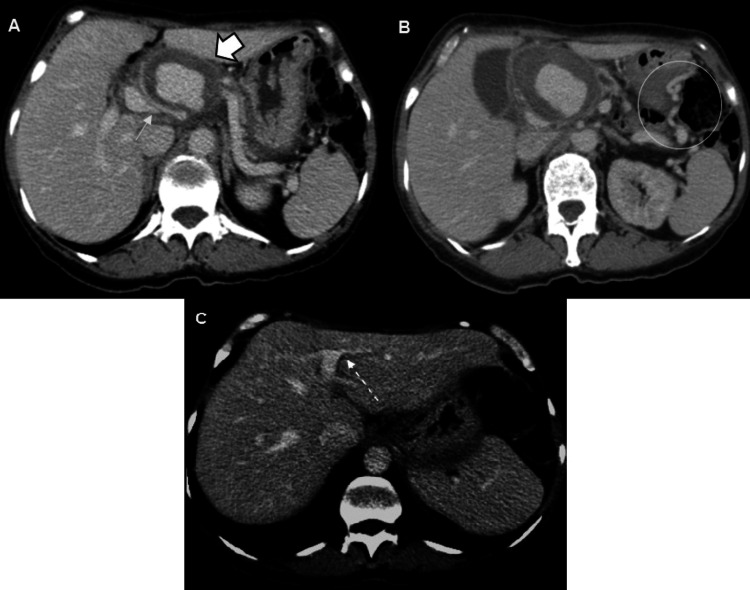
Computerized tomography of the abdomen in the venous portal phase. (A) Splenic artery pseudoaneurysm (thick arrow) causing a mass effect in the proximal region of the splenic vein, with a significant reduction in vessel caliber (thin arrow). (B) Multiple portosystemic collaterals secondary to splenic vein stenosis (white circle). (C) The pseudoaneurysm also causes mild dilation of the intrahepatic biliary ducts (dotted arrow).

This case presents a 55-year-old female patient with a history of recurrent pancreatitis. She was asymptomatic at the time, and an abdominal CT scan with intravenous contrast was requested for the follow-up of a pancreatic pseudocyst. In this scan, a rounded, thick-walled, partially defined image of heterogeneous density (average 40 HU) was observed adjacent to the hepatic hilum in a simple phase (Figure 1A), with homogeneous and central enhancement during contrast injection (average 123 HU; Figure 1B). It measured up to 6 cm in its largest diameter and is closely related to the splenic artery (Figure 2), compatible with pseudoaneurysm. This caused a mass effect in the body of the pancreas, resulting in dilation of the main pancreatic duct, the proximal region of the splenic vein with the formation of portosystemic collaterals (Figures 3A-3B), and the common bile duct, leading to dilation of the intrahepatic bile duct (Figure 3C).

## Discussion

A pseudoaneurysm is a vascular lesion that, unlike a true aneurysm, is contained only by a hematoma, surrounding tissue, or the fibrous reaction that develops around it [4]. Splenic artery pseudoaneurysms are extremely rare, with approximately 200 cases reported in the literature. The incidence is estimated at 0.1%-0.2% [5]. This represents a potentially fatal condition with a 37% risk of rupture and a 90% mortality rate following such an event [6].

This pathology is mainly caused by pancreatitis (56%), abdominal trauma (29%), and peptic ulcer disease (3%) [7]. An estimated 8% of cases are associated with chronic pancreatitis, as was the case described above [6]. Its origin is secondary to the interaction of the splenic artery with pancreatic enzymes, leading to necrotizing arteritis and destruction of the elastic tissues of the vessel wall, ultimately resulting in the formation of the pseudoaneurysm [5].

Although splenic artery pseudoaneurysms may be indolent and found as an incidental finding in imaging studies in up to 2.5% of cases, the vast majority present symptoms, with abdominal pain being the most common. Hematochezia, melena, and hematemesis are other symptoms that may be associated [5]. When there is a rupture, the most common is the sudden onset of intense abdominal pain and hemodynamic instability. Due to the high risk of this event, all pseudoaneurysms should be treated regardless of the symptoms [8].

It is well-established that angiography is the gold standard for diagnosing vascular diseases; however, in most cases, CT is the modality used to establish the diagnosis because it is the study of choice for detecting complications of pancreatitis. The sensitivity and specificity of CT angiography for identifying arterial complications in this context are 94% and 90%, respectively [9]. The characteristic imaging finding is a heterogeneous mass with central enhancement following contrast medium administration, closely associated with the splenic artery or pancreas. Its size typically ranges from 0.3 to 17 cm and may exert a mass effect on adjacent structures [5].

There are several modalities for the treatment of this disease based on the characteristics of the lesion and the patient. When it is located in the proximal third of the artery, a proximal and distal ligation of the vessel can be performed with subsequent excision of the pseudoaneurysm. If it is in the distal third, resection with splenectomy is preferred [5]. Transcatheter embolization is another technique to consider, which consists of catheterization of the splenic artery with subsequent embolization proximal and distal to the lesion with the use of coils achieving thrombosis of the pseudoaneurysm. Unlike surgical treatments, transcatheter embolization has a lower rate of complications and shortens the patient's hospital stay [8].

## Conclusions

Pseudoaneurysms are a rare complication of chronic pancreatitis, which has a high mortality rate if they rupture. Imaging studies are crucial for the diagnosis of this pathology, with CT being the most commonly used, especially in asymptomatic patients. Treatment is essential, and there are different modalities for this, with transcatheter embolization being one of the most commonly used currently, with a high success rate.
